# Investigation of the Anti-Inflammatory and Analgesic Activities of Ethanol Extract of Stem Bark of Sonapatha* Oroxylum indicum In Vivo*

**DOI:** 10.1155/2016/8247014

**Published:** 2016-01-26

**Authors:** K. Lalrinzuali, M. Vabeiryureilai, Ganesh Chandra Jagetia

**Affiliations:** Department of Zoology, Mizoram University, Aizawl 796004, India

## Abstract

Inflammation is all a pervasive phenomenon, which is elicited by the body in response to obnoxious stimuli as a protective measure. However, sustained inflammation leads to several diseases including cancer. Therefore it is necessary to neutralize inflammation. Sonapatha (*Oroxylum indicum*), a medicinal plant, is traditionally used as a medicine in Ayurveda and other folk systems of medicine. It is commonly used to treat inflammatory diseases including rheumatoid arthritis and asthma. Despite this fact its anti-inflammatory and analgesic effects are not evaluated scientifically. Therefore, the anti-inflammatory and analgesic activities of Sonapatha (*Oroxylum indicum*) were studied in Swiss albino mice by different methods. The hot plate, acetic acid, and tail immersion tests were used to evaluate the analgesic activity whereas xylene-induced ear edema and formalin induced paw edema tests were used to study the anti-inflammatory activity of Sonapatha. The administration of mice with 250 and 300 mg/kg b.wt. of* O. indicum* reduced pain and inflammation indicating that Sonapatha possesses analgesic and anti-inflammatory activities. The maximum analgesic and anti-inflammatory activities were observed in mice receiving 300 mg/kg b.wt. of* O. indicum* ethanol extract. Our study indicates that* O. indicum* possesses both anti-inflammatory and analgesic activities and it may be useful as an anti-inflammatory agent in the inflammation related disorders.

## 1. Introduction

The inflammation is a sequence of events that occurs in response to noxious stimuli, infection, trauma, or injury in the living tissues [1]. The inflammation is initiated by a cascade of events including enzyme activation, mediator release, fluid extravasations, cell migration, tissue breakdown, and repair processes [2]. The inflammation releases white blood cells as a protective measure against injury. These white blood cells synthesize several biomolecules and release them after injury leading to swelling and redness. The inflammation is characterized by induction of pain, redness, and rashes [3]. Prostaglandins are one of the important biomolecules, which play a key role in the induction of inflammatory response as their biosynthesis is significantly increased during inflammation [4]. The inflammatory responses are elicited as a defense mechanism by an organism or tissues; however, sustained inflammation can lead to undesired health effect as a consequence of interplay of various biomolecules that are secreted during the process of inflammation. Inflammation has been indicated in several diseases including cancer [5, 6]. The agents that contain or block inflammation may play an important role in treating pathologies associated with inflammatory reactions [7].

Natural products have a long history of use as a folk remedy for inflammatory conditions including fevers, pain, migraine, and arthritis. Many of the diseases in the modern world are thought to be due to inflammation; therefore, anti-inflammatory agents, anti-inflammatory food and food products are of great interest to contain or reduce inflammation-induced health disorders [8]. Fossil records indicate the use of natural products, especially the plants as medicine since Middle Paleolithic (approximately 60,000 years) age [9]. The modern allopathic drugs are single active chemical molecules and target one specific pathway, whereas herbal medicines contain pleiotropic molecules that work on an orchestral approach which are able to target many elements of the complex cellular pathway [10]. The pain and inflammatory conditions are usually managed by either steroidal (corticosteroids) or nonsteroidal (aspirin) drugs, which induce toxic side effects at different levels including allergic reactions, occasional hearing loss, and renal failure. These drugs also increase the risk of hemorrhage by negatively altering platelet function [11]. The medicinal plants have been a major source of a wide variety of biologically active compounds for many centuries and have been used extensively in crude form or as pure isolated compounds to treat various disease conditions including inflammation [12].

The inflammatory conditions can be cured using plant or plant derived products effectively.* Clerodendron inerme *has been reported to exhibit anti-inflammatory activity* in vitro* [13].* Hydrocotyle umbellata *and several other plants have been reported to possess anti-inflammatory activity in different study systems [14, 15]. Sonapatha or* Oroxylum indicum *belongs to Family Bignoniaceae and it is characterized by brown bark and large pinnate leaves. It is a medium sized, deciduous tree, distributed in India, Sri Lanka, Malaysia, China, Thailand, Philippines, and Indonesia. In India,* Oroxylum* is found in Eastern and Western Ghats and also in the North-East regions [16]. The existence of* Oroxylum indicum *(L) Vent. in natural population is highly threatened and it has been categorized as endangered medicinal plant by the Government of India. Various parts of* Oroxylum indicum* are utilized for medicinal purposes [17]. It has been used in Ayurveda and other traditional medicinal health systems since centuries [18]. The decoction of the bark is used to cure gastric ulcers and the bark paste is useful in treating mouth cancer, scabies, and other skin diseases. The bark paste is applied to the wounds of animals to kill maggots. Poultice of the bark is topically applied to treat rheumatism, sprains, inflammations, and skin diseases [19]. The bark decoction of* Oroxylum* is also a useful remedy to deworm cattle [20]. Apart from this,* Oroxylum* species are reported to have a variety of medicinal properties like anticancer, antiulcer, antidysenteric, antimicrobial, and anti-inflammatory [21]. It has been shown to be antibacterial, antioxidant, hepatoprotective, and immunomodulatory [22]. From the above it is clear that the systematic evaluation of anti-inflammatory and analgesic activities of* Oroxylum indicum* is lacking, which stimulated us to obtain an insight into the anti-inflammatory and analgesic activities of* Oroxylum indicum *in Swiss albino mice.

## 2. Materials and Methods

### 2.1. Preparation of Extract

The noninfected and matured stem bark of* Oroxylum indicum *(Family: Bignoniaceae) was collected from Champhai (23.456°N latitude and 93.329°E longitude), Mizoram, India, during the month of January. The plant was identified by the Department of Horticulture and Aromatic and Medicinal Plants, Mizoram University, Aizawl, India. The bark of* O. indicum *was thoroughly rinsed with clean water and shade dried at room temperature in the dark in clean and hygienic conditions. The dried bark was powdered in an electrical grinder at room temperature. The stem bark powder of* O. indicum *was sequentially extracted in petroleum ether, chloroform, ethanol, and distilled water according to increase in polarity using a Soxhlet apparatus until the solvents became colourless [23]. The ethanol extract was concentrated using rotary evaporator and stored at −70°C until further use. Henceforth the ethanol extract of* O. indicum* will be referred to as OIE throughout the paper.

### 2.2. Animal Care and Handling

The animal care and handling were carried out according to the guidelines issued by the World Health Organization, Geneva, Switzerland, and the INSA (Indian National Science Academy, New Delhi, India). Usually, 6-to-8-week-old healthy male Swiss albino mice weighing 30–35 g were selected from an inbred colony maintained under the controlled conditions of temperature (25 ± 2°C) and humidity (55–60%) with 12 hours of light and dark cycle, respectively. The animals were housed in a sterile polypropylene cage containing paddy husk (procured locally) as bedding material. The animals had free access to standard rodent diet and water. All animal experiments were carried out according to NIH and Indian National Science Academy, New Delhi, India guidelines. The study was approved by the Institutional Animal Ethics Committee of the Mizoram University, Aizawl, Mizoram: India vide letter number MZU/IAEC/4503.

### 2.3. Experimental

The anti-inflammatory and analgesic activities were determined by dividing the animals into the following groups.

#### 2.3.1. SPS Group

The animals of this group did not receive any treatment except the sterile physiological saline (SPS).

#### 2.3.2. DIF Group

The animals of this group were injected with 20 mg/kg b.wt. of diclofenac sodium intraperitoneally.

#### 2.3.3. OIE Group

The animals of this group were administered with 250 and 300 mg/kg b.wt. of ethanol extract of* Oroxylum indicum* intraperitoneally.

The analgesic and anti-inflammatory activities were determined 30 minutes after the administration of SPS or diclofenac (DIF) or ethanol extract of* Oroxylum indicum*.

### 2.4. Analgesic Activity

The analgesic activity of OIE was determined by carrying out the following tests.

#### 2.4.1. Hot Plate Test

A separate experiment was conducted to determine analgesic activity of OIE by the hot plate test as described earlier [24], where the grouping and other conditions were essentially similar to those described above. The hotplate contained metallic surface (diameter 20 and 10 cm high) and its temperature was set at 55°C. Briefly, each mouse was placed onto the hotplate and covered with a glass beaker to avoid heat loss. Each mouse also acted as its own control. The time taken to lick the fore paws or jumping was recorded. The latency is defined as the reaction time taken by each mouse to respond to licking of the fore paws or jumping. Untreated animals exhibiting latency of 5–20 s were selected. The latency period for all groups was recorded thirty minutes after administration. Usually 10 mice were used for each group.

The percent inhibition was calculated as follows:

(1) Posttreatment  latencys−Pretreatment  latencys×100Pre-treatment  latencys.

#### 2.4.2. Acetic Acid Induced Writhing Test

A separate experiment was performed to evaluate the analgesic activity by acetic acid induced writhing test, which was carried out as described earlier [25]. The grouping and other conditions were essentially similar to those described earlier. The mice were administered intraperitoneally with 0.7% v/v acetic acid (volume of acetic acid did not exceed 10 *μ*L/g b.wt.). Immediately after acetic acid administration, the mice were individually placed into glass beakers and five min was allowed to elapse. The number of writhes produced in these animals was counted up to 30 min. For scoring purposes, a writhe is indicated by stretching of the abdomen with simultaneous stretching of at least one hind limb. Usually 10 mice were used for each group.

Inhibition of writhing (%) was calculated as follows:

(2) $$\begin{array}{c} \frac{\text{Control}-\text{Treated}}{\text{Control}}\times 100. \end{array}$$

#### 2.4.3. Tail Immersion Test

A separate experiment was conducted to evaluate the analgesic activity of Sonapatha ethanol extract by tail immersion test according to the procedure described elsewhere [26]. The grouping and other conditions were essentially similar to those described above in Section 2.3. The tail immersion test was carried out in a hot water bath set at a temperature of 55 ± 0.5°C, where 3 cm of animal tail was immersed into the hot water and tail withdrawal reaction was recorded as time in seconds in all groups using a digital stopwatch. A minimum of two observations were collected for each animal in control group, immediately and 10 min after the initial reading. The tail withdrawal test was carried out in the treatment groups periodically at 0, 0.5, 1, 2, 3, 4, and 6 hours after administration of OIE, or diclofenac. Usually 10 mice were used for each group.

### 2.5. Anti-Inflammatory Activity

The anti-inflammatory activity was studied by xylene-induced ear edema and formalin induced paw edema in mice.

#### 2.5.1. Xylene-Induced Ear Edema

A separate experiment was carried out to evaluate the anti-inflammatory activity by xylene-induced ear edema as described earlier [27]. The grouping and other conditions were essentially similar to those described above in Section 2.3. Mice were divided into five groups of 10 animals each. The mice were intraperitoneally administered with either distilled water (10 *μ*L/g b.wt.) or diclofenac (20 mg/kg b.wt.) or OIE (250–300 mg/kg b.wt.). Thirty minutes after administration the inner surface of right ear of each animal was applied with 0.03 mL of xylene for the induction of ear edema, whereas the left ear served as the control. Fifteen minutes after the application of xylene, the mice were killed under ketamine anesthesia. Circular sections of both the ears were taken, using a cork borer of 6 mm, and weighed:

(3) $$\begin{array}{c} \text{Inhibition (\%)}=\frac{\text{Difference in ear weight (control)}-\text{Difference in ear weight (test)}}{\text{Difference in ear weight (control)}}\times 100. \end{array}$$

#### 2.5.2. Formalin Induced Inflammation

A separate experiment was conducted to evaluate the anti-inflammatory activity by formalin induced inflammation. The grouping and other conditions were essentially similar to those described above in Section 2.3. The anti-inflammatory activity was assessed as described earlier [28]. Swiss albino mice were divided into groups of ten. The inflammation was produced by subaponeurotic injection of 0.1 mL of 2% formaldehyde in the right hind paw of the mice on the first and third day. The animals were treated daily with the OIE and diclofenac intraperitoneally for 10 days. The daily changes in paw size were measured by wrapping a piece of cotton thread around the paw and measuring the circumference with a meter rule. Usually 10 mice were used for each group.

### 2.6. Statistical Analysis

The data were analyzed by one-way ANOVA, followed by application of Tukey test (Pro 8 SRO v8.0724 (B724)), Northampton, MA, USA. A *P* value of <0.05 was considered to be statistically significant.

## 3. Results

**Figure 1:**
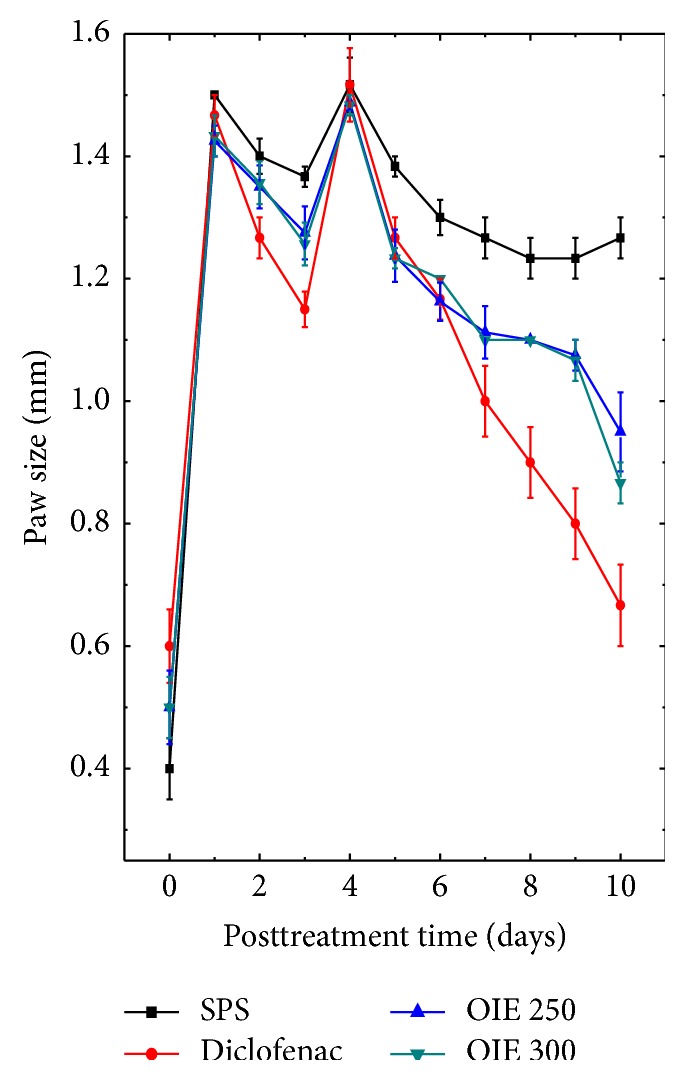
Effect of ethanol extract of* Oroxylum indicum* on the formalin induced inflammation in mice paw.

The results of analgesic and anti-inflammatory activities are presented in Tables 1–4 and Figure 1.

### 3.1. Analgesic Activity

#### 3.1.1. Hot Plate Test

The analgesic activity was assessed using the hot plate method (Table 1). The administration of ethanol extract (OIE) showed a significant analgesic activity for both 250 and 300 mg/kg and 300 mg/kg exhibited the highest activity (62.5% inhibition) as compared to 250 mg/kg b.wt. (52.63% inhibition) as indicated by pain attenuation. The positive control diclofenac showed higher analgesic activity than OIE (76.31% inhibition) at a dose of 20 mg/kg b.wt. (Table 1).

#### 3.1.2. Acetic Acid Induced Writhing Test

The results of acid writhing test are depicted in Table 2. Administration of acetic acid to control mice produced 66.2 ± 1.16 writhes within 30-minute observation period. Pretreatment with the OIE at 250 and 300 mg/kg b.wt. reduced the number of writhes up to 22.5 ± 1.12 (66.0% inhibition) and 20.8 ± 0.74 (68.58% inhibition), respectively. The standard drug diclofenac reduced the number of writhes to 10.8 ± 0.74 (83.68% inhibition) at a dose of 20 mg/kg b.wt. (Table 2).

#### 3.1.3. Tail Immersion Test

Analgesic activity was also estimated using tail immersion test. The tail immersion test indicated that both the doses of the extract as well as the positive controls showed a significant inhibition in tail immersion rest when compared to the negative control (Table 3). The maximum analgesic effect was recorded for 300 mg/kg b.wt. OIE. The diclofenac (positive control) showed better activity as compared to the OIE.

### 3.2. Anti-Inflammatory Activity

#### 3.2.1. Xylene-Induced Ear Edema

The results of the anti-inflammatory study using xylene-induced ear edema are shown in Table 4. SPS treated control mice showed an increase in ear weight up to 13.98 ± 0.60 mg and the OIE administration has inhibited this weight gain by 68.097% (4.46 ± 0.89 mg) and 71.3877% (4.00 ± 0.24 mg) for 250 and 300 mg/kg b.wt. of OIE, respectively (Table 4). The positive control diclofenac showed 52.72% (6.61 ± 0.49 mg) inhibition at 20 mg/kg b.wt. which was lower as compared to both the doses of OIE (Table 4).

#### 3.2.2. Formalin Induced Inflammation

Treatment of mice with OIE gradually reduced diameters of the paw with time in both the treated and positive control groups (Figure 1). The OIE reduced the inflammatory reactions when compared to the SPS control group as indicated by the significant reduction in the paw diameter (Figure 1). However, the effect was more pronounced for 300 mg/kg OIE treatment (Figure 1).

## 4. Discussion

Inflammation is well orchestrated response to deleterious stimuli including tissue injury, and infection [29]. It is elicited to restore normal condition of tissue or body. Classically inflammation is characterized by increase in the blood flow, reddening of the affected part due to increased erythrocyte accumulation and edema [30]. Physiologically inflammation results in the secretion of numerous cytokines, acute phase proteins, and recruitment of leucocytes to the site of injury [29]. Inflammation has been indicated as a major cause in the development of several diseases in humans including neurological, cardiovascular, intestinal, dental, and renal disorders. Inflammation is also linked to ageing, diabetes, obesity, ankylosing spondylitis, multiple sclerosis, pancreatitis, and cancer [29, 31–36]. The strategies to combat inflammation will be useful in reducing the inflammation related disorders. Therefore, the present study was undertaken to evaluate the analgesic and anti-inflammatory activities of* Oroxylum indicum* in mice.

The analgesic activity of* Oroxylum indicum* was studied by the hot plate, tail immersion, and acetic acid tests, which are standard procedures to evaluate central and peripheral nervous system acting analgesics [37, 38]. The acetic acid is known to trigger the production of noxious substances within the peritoneum resulting in writhing response [37]. It is a simple, rapid, and reliable model and especially suitable to evaluate peripheral type of analgesic action of any drug [39]. The administration of* Oroxylum indicum* extract showed a significant analgesic activity indicating that it has some analgesic effect on both the central and peripheral nervous systems as indicated by reduced pain by hot plate method and suppression of acetic acid induced writhing. Several plant extracts including* Adhatoda vasica*,* Acacia hydaspica*,* Boswellia serrate*,* Glaucium grandiflorum*, and* Landolphia owariensis *have shown analgesic activity* in vivo* [40–44]. The tail immersion test has been used as a standard procedure to study the analgesic activity of pharmacological agents [45, 46], which was originally devised by [47]. The withdrawal latency is unusually determined once or twice to limit the conditioning effect [46]. The increase in the tail withdrawal latency is a good measure of analgesia induced by any chemical agent. Treatment of mice with* Oroxylum indicum* extract increased tail withdrawal latency confirming its analgesic effects.

The anti-acute inflammatory activity of any agent can be determined by xylene-induced ear edema or formalin induced paw edema tests [48, 49]. The formalin administration elicits behavioral effects stimulated by nociceptors. The inflammatory phase induced pain evokes a combination of stimuli, including inflammation of peripheral tissues and mechanisms of central sensitization [50, 51]. The central nervous system acting drugs including opioids suppress both phases equally; however drugs that act on peripheral nervous system such as NSAIDs and corticosteroids only inhibit the second phase [50]. Our findings indicate that* Oroxylum indicum* extract acts as anti-inflammatory agent as it reduced the xylene-induced ear edema as well as formalin induced paw edema in treated mice.* Oroxylum indicum* extract has been effective in both the central and peripheral nervous systems since it was able to desensitize neurons of both central and peripheral nervous systems equally as indicated by the attenuation of pain and inflammation. Many plants have been reported to possess anti-inflammatory activity in various study systems [15, 52]. Similarly,* Adhatoda vasica*,* Acacia hydaspica*,* Boswellia serrate*,* Glaucium grandiflorum*, and* Landolphia owariensis*,* Harpagophytum procumbens*,* Rosa canina*,* Oenothera biennis*,* Ribes nigrum*,* Borago officinalis*,* Zingiber officinale*,* Nigella sativa*, and* Folium eriobotryae *have been reported to act as anti-inflammatory agents in different study systems [40–44, 53, 54].

The exact mechanism of suppression of inflammation by* O. indicum* is not known. However, it contains flavonoids and other phenolic compounds that may have contributed to its analgesic and anti-inflammatory actions.* O. indicum* has been found to scavenge DPPH, superoxide anion, hydroxyl, nitric oxide, and Fe^3+^ radicals, which are major players in eliciting the inflammatory response [55]. Apart from free radical scavenging, it may have also reduced activation of cytokines like NF-*κ*B, TNF*α*, IL-1*β*, and IFN*γ*. Inflammation has been reported to stimulate the activation of these cytokines [56, 57]. Biochanin-A present in the root bark of* O. indicum* has been reported to inhibit TNF*α* [58]. Chrysin has been isolated from the ethanol extract of* O. indicum* (data not shown) which has been reported to suppress the transcriptional activation of NF-*κ*B and Cox-II [59]. The observed anti-inflammatory action of* O. indicum* may also be due to its inhibitory action on cyclooxygenase which is involved in prostaglandin synthesis [60].

## 5. Conclusions

Our study demonstrates that the* O. indicum* acts as an analgesic and anti-inflammatory agent. The analgesic and anti-inflammatory activities of* O. indicum* may be due to its ability to neutralize free radicals which are the main players in inflammation. It may have also suppressed the activation of proinflammatory cytokines including NF-*κ*B, TNF*α*, IL-1*β*, and IFN*γ* and the activity of cyclooxygenase enzymes which are involved in inflammation. The anti-inflammatory and analgesic activities of* O. indicum* may be due to the presence of flavonoids and other polyphenols. The* O. indicum* may be used to reduce inflammation; however, further studies are required to understand molecular mechanisms of action against inflammation.

## Figures and Tables

**Table 1:** Effect of *Oroxylum indicum* on the analgesic activity in mice by hot plate test.

Treatment	Dose (mg/kg b.wt.)	Mean ± SEM		Increase in latency period (%)
		Pretreatment reaction latency(s)	Posttreatment reaction latency(s)	
Control	0	7.60 ± 0.58	7.60 ± 0.45	0
OIE	300	8.00 ± 0.32	13.20 ± 1.07^*∗*^	62.5
OIE	250	7.60 ± 0.89	11.60 ± 1.04^*∗*^	52.63
Diclofenac	20	7.60 ± 0.55	13.40 ± 0.84^*∗*^	76.31

*N* = 10.

^*∗*^ *P* < 0.05 when compared to SPS treated control.

**Table 2:** Alteration in the analgesic activity by acetic acid induced writhing in mice treated with different doses of* Oroxylum indicum*.

Treatment	Dose (mg/kg b.wt.)	Mean ± SEM	Percentage inhibition of writhing (%)
		Number of writhes	
Control	0	66.2 ± 1.16	0
OIE	300	20.8 ± 0.74^*∗*^	68.58
	250	22.5 ± 1.12^*∗*^	66.01
Diclofenac	20	10.8 ± 0.74^*∗*^	83.68

*N* = 10.

^*∗*^ *P* < 0.05 when compared to SPS treated control.

**Table 3:** Alteration in the response time in mice treated with *Oroxylum indicum *before subjecting them to tail immersion test.

Treatment	Dose (mg/kg b.wt.)	Response time in seconds ± SEM							
		Assessment time (h)							
		0	0.5	1	2	3	4	5	6
Control	0	4.30 ± 0.05	4.2 ± 0.20	4.03 ± 0.12	4.6 ± 0.35	4.81 ± 0.51	4.62 ± 0.42	4.31 ± 0.37	4.30 ± 0.40

OIE	300	5.67 ± 0.20	6.33 ± 0.15^*∗*^	6.78 ± 0.51^*∗*^	8.80 ± 0.06^*∗*^	7.56 ± 0.05^*∗*^	6.98 ± 0.15^*∗*^	6.80 ± 0.20^*∗*^	6.61 ± 0.27^*∗*^
			(11.64)	(19.57)	(55.20)	(33.33)	(23.10)	(19.92)	(16.57)
	250	4.69 ± 0.50	4.72 ± 0.08^*∗*^	4.97 ± 0.30^*∗*^	4.78 ± 0.46^*∗*^	7.14 ± 0.54^*∗*^	5.65 ± 0.65^*∗*^	4.90 ± 0.15^*∗*^	4.80 ± 0.10^*∗*^
			(1.91)	(5.97)	(19.18)	(52.23)	(20.46)	(4.47)	(2.35)

Diclofenac	20	4.29 ± 0.08	4.43 ± 0.20^*∗*^	5.03 ± 0.11^*∗*^	6.76 ± 0.5^*∗*^	7.31 ± 0.57^*∗*^	6.87 ± 0.48^*∗*^	6.39 ± 0.47^*∗*^	4.50 ± 0.70^*∗*^
			(3.26)	(17.29)	(57.57)	(70.39)	(60.13)	(48.95)	(4.89)

Inhibition (%) is shown in brackets.

*N* = 10.

^*∗*^ *P* < 0.05 when compared to SPS treated control.

**Table 4:** Effect of ethanol extract of *Oroxylum indicum *on xylene-induced ear edema in mice.

Treatment	Dose (mg/kg b.wt.)	Mean increase in ear weight (mg) ± SEM	% Inhibition
Control	0	13.98 ± 0.60	—
OIE	300	4.00 ± 0.24^*∗*^	71.39
	250	4.46 ± 0.89^*∗*^	68.10
Diclofenac	20	6.61 ± 0.49^*∗*^	52.72

*N* = 10.

^*∗*^ *P* < 0.05 when compared to SPS treated control.
